# Cryo-EM structure of the RuvAB-Holliday junction intermediate complex from *Pseudomonas aeruginosa*

**DOI:** 10.3389/fpls.2023.1139106

**Published:** 2023-03-21

**Authors:** Xu Zhang, Zixuan Zhou, Lin Dai, Yulin Chao, Zheng Liu, Mingdong Huang, Qianhui Qu, Zhonghui Lin

**Affiliations:** ^1^ College of Chemistry, Fuzhou University, Fuzhou, China; ^2^ Shanghai Stomatological Hospital, Institutes of Biomedical Science, Department of Systems Biology for Medicine, Fudan University, Shanghai, China; ^3^ Kobilka Institute of Innovative Drug Discovery, School of Medicine, Chinese University of Hong Kong, Shenzhen, China

**Keywords:** homologous recombination, DNA damage repair, Holliday junction, RuvA, RuvB, complex assembly, branch migration, *Pseudomonas aeruginosa*

## Abstract

Holliday junction (HJ) is a four-way structured DNA intermediate in homologous recombination. In bacteria, the HJ-specific binding protein RuvA and the motor protein RuvB together form the RuvAB complex to catalyze HJ branch migration. *Pseudomonas aeruginosa* (*P. aeruginosa*, Pa) is a ubiquitous opportunistic bacterial pathogen that can cause serious infection in a variety of host species, including vertebrate animals, insects and plants. Here, we describe the cryo-Electron Microscopy (cryo-EM) structure of the RuvAB-HJ intermediate complex from *P. aeruginosa*. The structure shows that two RuvA tetramers sandwich HJ at the junction center and disrupt base pairs at the branch points of RuvB-free HJ arms. Eight RuvB subunits are recruited by the RuvA octameric core and form two open-rings to encircle two opposite HJ arms. Each RuvB subunit individually binds a RuvA domain III. The four RuvB subunits within the ring display distinct subdomain conformations, and two of them engage the central DNA duplex at both strands with their C-terminal β-hairpins. Together with the biochemical analyses, our structure implicates a potential mechanism of RuvB motor assembly onto HJ DNA.

## 1 Introduction

Homologous recombination (HR) is a fundamental biological process responsible for genetic diversity generation and DNA damage repairs in all three domains of life (Cromie et al., 2001). During DNA double-strand break (DSB) repair by HR, two homologous duplex DNA molecules intersect each other, forming a four-way structured recombination intermediate termed Holliday junction (HJ) (Holliday, 1964). These HJ structures need to be properly processed to complete the DNA damage repairs (San Filippo et al., 2008; Heyer et al., 2010; Matos and West, 2014; Wyatt and West, 2014).

In bacteria, the RecG and RuvABC proteins have been implicated in the processing of HJ intermediates. RecG belongs to the superfamily 2 (SF2) DNA helicase and has been shown to be required for HJ intermediate formation (Singleton et al., 2001; Mawer and Leach, 2014), while the SOS-inducible proteins RuvA and RuvB work in concert with RuvC to catalyze HJ branch migration and resolution (Shinagawa and Iwasaki, 1996; West, 1996; West, 1997). Previous structural studies have revealed that RuvA is a HJ-specific binding protein (Parsons et al., 1995), and can either bind HJ as a single tetramer or sandwich HJ with two tetramers (Rafferty et al., 1996; Hargreaves et al., 1998; Ariyoshi et al., 2000). RuvB belongs to the ring-shaped helicase superfamily, which can be divided into two major categories, the RecA-like ATPase and AAA+ ATPase (Singleton et al., 2007; O'Donnell and Li, 2018; Gao and Yang, 2020). Members of this family have been shown to be involved in various fundamental cellular processes, such as DNA replication (Hamdan and Richardson, 2009; Gao et al., 2019), RNA synthesis (Nogales et al., 2017), homologous recombination and DNA repair (Yamada et al., 2004; Bizard and Hickson, 2014; Clapier et al., 2017). During homologous recombination, the RuvB hexameric motors are attached to the RuvA-HJ core complex through the interactions with RuvA domain IIIs (RuvA^D3^) and thereby drive HJ branch migration and strand exchange between the two homologous DNAs (Iype et al., 1994; Iype et al., 1995; Adams and West, 1996; Wald et al., 2022).

The gram-negative bacterium *Pseudomonas aeruginosa* (*P. aeruginosa*, Pa), widely thrives in natural environments, is an opportunistic pathogen of a variety of host species, including vertebrate animals, insects and plants (Mahajan-Miklos et al., 1999; Jander et al., 2000; Walker, 2004). For example, the highly virulent clinical strains PA14 and PAO1 have been shown to infect the roots of *Arabidopsis thaliana* and sweet basil (*Ocimum basilicum*) and can cause plant mortality (Walker et al., 2004). The emergence and development of multidrug-resistance has raised *P. aeruginosa* to the first critical group of the WHO list of resistant to antibiotic ‘priority pathogens’. Genomics studies have revealed that antibiotic rsistance can be acquired by HR-mediated horizontal gene transfer (Eisen, 2000). Therefore, the RuvAB proteins have been recognized as attractive targets to combat *P. aeruginosa* infections. We recently reported that the small-molecule inhibitors of PaRuvAB could increase the susceptibility of *P. aeruginosa* to UV-C irradiation (Dai et al., 2022).

In the present work, we describe the structure of the RuvAB-HJ complex from *P. aeruginosa* by cryo-EM. The complex contains eight RuvA subunits, eight RuvB subunits and one HJ molecule. The eight RuvA subunits form two tetramers sandwiching HJ at the junction center as previously described (Roe et al., 1998; Wald et al., 2022). The eight RuvB subunits constitute two open-rings encircling two opposite HJ arms, with each subunit binding to a RuvA^D3^. Together with the biochemical analyses, our structure suggests an intermediate state of RuvAB-HJ complex, and implicates a potential mechanism of RuvB motor assembly onto HJ DNA.

## 2 Results

### 2.1 Reconstitution of the RuvAB-HJ complex

**Figure 1:**
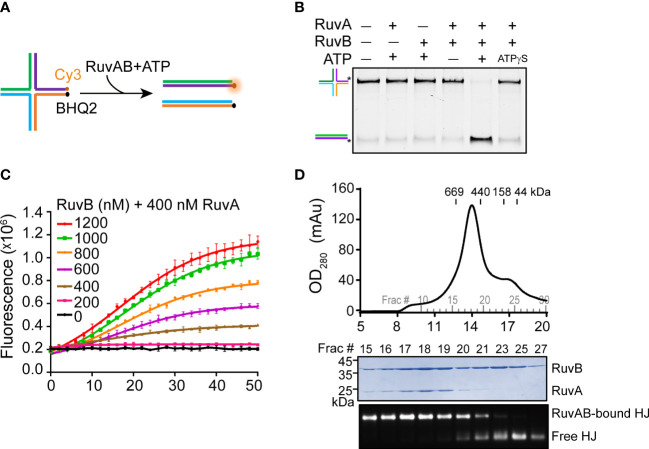
Reconstitution of the PaRuvAB-HJ complex. **(A)** Schematic representation of HJ branch migration by the PaRuvAB complex assayed by the FRET-based duplex unwinding assay. The complete sequences are listed in **Supplementary Table S1**. HJ strands are labeled with Cy3 fluorophore and BHQ2 quencher as indicated. **(B)** The native polyacrylamide gel analysis of HJ DNA unwinding by the RuvA and RuvB proteins. HJ strands are color-coded as in **(A)**, and fluorescently labelled with 6-carboxyfluorescein (FAM) as indicated by asterisks (*). **(C)** Real-time monitoring of branch migration activities of RuvA (400 nM) with increasing amounts of RuvB. The fluorescent signal was recorded every 2 min (mean ± s.d., n = 3 independent experiments). **(D)** Size-exclusion chromatography of the PaRuvAB-HJ complexes (top panel) and Coomassie-stained gel of proteins and agarose gel of HJ-DNA eluted from the column (bottom panel).

To reconstitute the RuvAB-HJ complex, we prepared a synthetic HJ by annealing four 55-bp DNA strands (**Figure 1A**; **Supplementary Table S1**). The recombinant PaRuvA and PaRuvB proteins were separately expressed and purified from bacterial cells (**Supplementary Figure S1**). We first tested whether the purified recombinant proteins were active in HJ branch migration. In the gel-based DNA unwinding assay, PaRuvA in combination with PaRuvB, but not each alone, could unwind synthetic HJs into two linear DNA duplexes in the presence of ATP (**Figure 1B**). To quantitatively monitor this reaction in real time, we then performed a fluorescence resonance energy transfer (FRET) assay using the Cy3/BHQ2 modified-HJ as substrate (**Figure 1A**). At a fixed amount of PaRuvA (400 nM), the fluorescence intensity was gradually enhanced with increasing concentrations of PaRuvB, and reached a plateau at 1000 nM (**Figure 1C**). These data thus suggest that the recombinant PaRuvAB complex is active in HJ branch migration.

We next sought to reconstitute the PaRuvAB-HJ complex for cryo-EM. For more than 30 years efforts, researchers have determined the structures of RuvAB (Yamada et al., 2002), RuvA-HJ (Rafferty et al., 1996; Hargreaves et al., 1998; Ariyoshi et al., 2000), but have not obtained the atomic structure of RuvAB-HJ tripartite complex, presumably due to the labile nature of the reconstituted complexes. Until recently, while we were preparing this manuscript, Wald et al. broke the stalemate by making a hetero-RuvAB complex (RuvA from *Salmonella typhimurium* and RuvB from *Streptococcus thermophilus*), which turned out to be more stable than the homo-complexes (Wald et al., 2022). In this study, we first incubated PaRuvA with HJ DNA at 4°C for 15 min with a molar ratio of 8:1.2 to allow the formation of RuvA-HJ core complex. Then, the mixture was further incubated with excess PaRuvB proteins (RuvB:RuvA = 2:1) at 4°C for another 15 min. The resulting mixture was further purified through the superose 6 size-exclusion column. The peak eluted at ~13.9 ml contained both PaRuvA and PaRuvB proteins and HJ DNA (**Figure 1D**). However, the molecular weight (MW) of this complex was much smaller than the predicted MW (712 kDa) based on the retention times of a gel filtration standard (**Figure 1D**), indicating that this might be an intermediate complex. Since we could not reconstitute the intact RuvAB-HJ complex, we therefore focused on the intermediate complex for cryo-EM structure determination.

### 2.2 Cryo-EM and overall structure of the RuvAB-HJ intermediate complex

**Figure 2:**
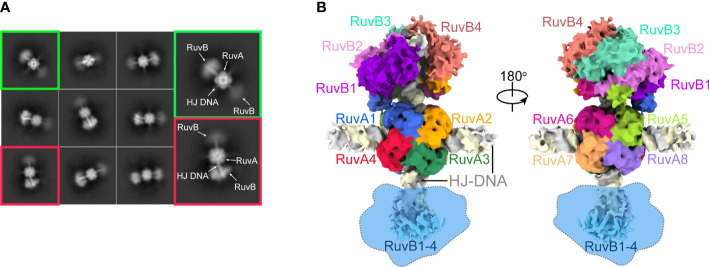
Cryo-EM of the PaRuvAB-HJ complex. **(A)** Representative 2D classes of the PaRuvAB-HJ complex. **(B)** Cryo-EM map for the PaRuvAB-HJ complex. The components within the complex are labeled and colored as indicated. The dashed blobs indicate the less resolved map of the other RuvB motor. All structural figures in this study, unless otherwise indicated, use the same color and labeling schemes.

We next performed cryo-EM experiment using the above purified RuvAB-HJ tripartite complex. After imaging processing and 2D classification, about 551,160 particles of dumbbell shape were selected for 3D reconstruction and refinement (**Figure 2A**; **Supplementary Figure S2**). As a result, a cryo-EM map of the PaRuvAB-HJ intermediate complex was obtained at an overall resolution of ~ 6.0 Å, and the local resolutions for the PaRuvA-HJ core and PaRuvB-HJ arms were estimated to be ~ 3.0 Å and 7.0 Å, respectively (**Supplementary Figure S2**, **S3**). In addition, the results of 3D variability analysis of cryo-EM map revealed multiple conformations in the RuvB moiety, implying that there might be variable catalytic states in the sample (**Supplementary movie S1**).

The PaRuvA-HJ model was built based on the crystal structure of *Escherichia coli* (*E. coli*, Ec) RuvA-HJ complex (PDB ID: 1C7Y)(Ariyoshi et al., 2000). It clearly shows two RuvA tetramers sandwiching one HJ DNA molecule (**Figure 2B**), as described in the previous crystal structures of RuvA-HJ complexes (Roe et al., 1998). For PaRuvB-dsDNA complex model building, we determined a 2.1-Å crystal structure of PaRuvB in complex with ADP (**Supplementary Table S2**). The resulting PaRuvA-HJ and PaRuvB-dsDNA structures were subsequently used to composite the model of PaRuvAB-HJ tripartite complex. Owing to the resolution limits of current optimization methods, the final model only shows single RuvB motor (**Figure 2B**), although the particles selected for map reconstruction clearly showed two RuvB motors within a single complex (**Figure 2A**). Moreover, because RuvAB catalyzes HJ branch migration in a non-sequence-specific manner, and the density for the nucleobases is the averaging result of all four possibilities, no sequence was assigned to HJ DNA in the structures.

### 2.3 Structure of the PaRuvA-HJ core complex

**Figure 3:**
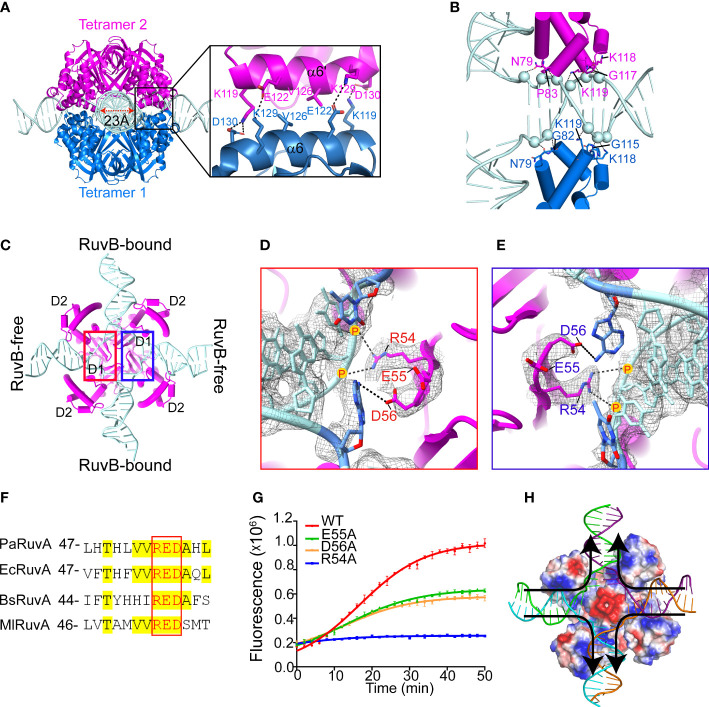
Cryo-EM structure of the PaRuvA-HJ complex. **(A)** Dimerization interface between the two RuvA tetramers, with defined region magnified and shown on the right. RuvA is shown as cartoon representation and HJ is shown as ladder. Key interacting residues are shown in sticks. **(B)** Zoomed in view of the interaction between HJ duplex arm and RuvA^D2^ from two separate tetramers, with corresponding residues shown in sticks and DNA backbone phosphates shown in spheres. **(C)** An interior view of RuvA-HJ complex. **(D, E)** A close view of the defined regions in **(C)**. RuvA RED motifs within the tetramer are shown in sticks. DNA is shown in ladder representation. 2mFo-DFc map is contoured at 0.38 σ. **(F)** Sequence alignment of the RED motifs of RuvA from various species. Pa, *Pseudomonas aeruginosa;* Ec, *Escherichia coli;* Bs, *Bacillus subtilis;* Ml, *Mycobacterium leprae.* **(G)** Effects of RED motif mutations on the branch migration activity of RuvAB complex by FRET-based assay (mean ± s.d., n = 3 independent experiments). **(H)** Cartoon diagram of HJ branch migration. RuvA tetramer is shown as surface and colored with its electrostatic potential (red, negative; blue, positive; white, neutral). DNA is shown as ladder. Arrows indicated the direction of branch migration.

The cryo-EM structure of PaRuvA-HJ region was determined at 3.0 Å with well-defined electron density (**Supplementary Figure S4A**). In agreement with the previous crystal structures of HJ-bound octameric RuvA from *Mycobacterium leprae* (*M. leprae*, Ml) (Roe et al., 1998), each PaRuvA subunit is composed of three distinct domains: D1, D2 and D3 (**Supplementary Figure S4B**). The HJ molecule is sandwiched by two PaRuvA tetramers that dimerize through a pair of antiparallel helices, involving at least four pairs of electrostatic interactions and one pair of hydrophobic interaction (**Figure 3A**). A triple mutation (E122K/V126A/D130K) at the dimerization interface significantly diminished the branch migration activity of PaRuvAB complex (**Figure 3A** and **Supplementary Figure S4C**), implying that the formation of RuvA octamer is required for efficient branch migration. The two tetramers together enclose a cross-shaped tunnel of ~ 23 Å in diameter, which encircles ~ 10 bp of HJ arm (**Figure 3A**; **Supplementary Figure S4D, E**). The protein-DNA contacts at each HJ arm are mainly mediated by the RuvA^D2^ and DNA phosphodiester backbone (**Figure 3B**). Interestingly, all RuvA^D2^ in tetramer 1 insert into the DNA major groove, whereas the equivalent domains in tetramer 2 all bind the minor groove (**Figure 3B**). As a result, the two tetramers do not form a perfect two-fold symmetry but are misaligned by ~18° (**Supplementary Figure S4E**).

The junction center is devoid of base pairs but occupied by a conserved ‘RED’ motif (R54, E55 and D56) in RuvA^D1^ (**Figures 3C–F**). In the motif, R54 binds to the ribose-phosphate backbone of the branch site with its positively charged guanidino group, while the carboxyl groups of E55 and D56 may provide strong negatively electrostatic repulsion for strand separation (**Figures 3D, E**). Of note, the base pairs at the branch sites of RuvB-free HJ arms are disrupted and interact with D56, whereas the equivalent base pairs at RuvB-bound arms remain intact (**Figures 3C–E**). Alanine substitution of R54 completely abolished the HJ branch migration activity, while the E55A and D56A mutations caused ~ 50% reductions (**Figure 3G**). These observations thus suggest that base pairs passing through the acidic center would be disrupted through the transient interactions with RED motifs, and after that, new base pairs would form once the DNA duplex are pumped out by RuvB motor through the exit gates (**Figure 3H**). This is the basis of DNA strand exchange at the HJ branch migration phase.

### 2.4 Recruitment and assembly of the RuvB motor

**Figure 4:**
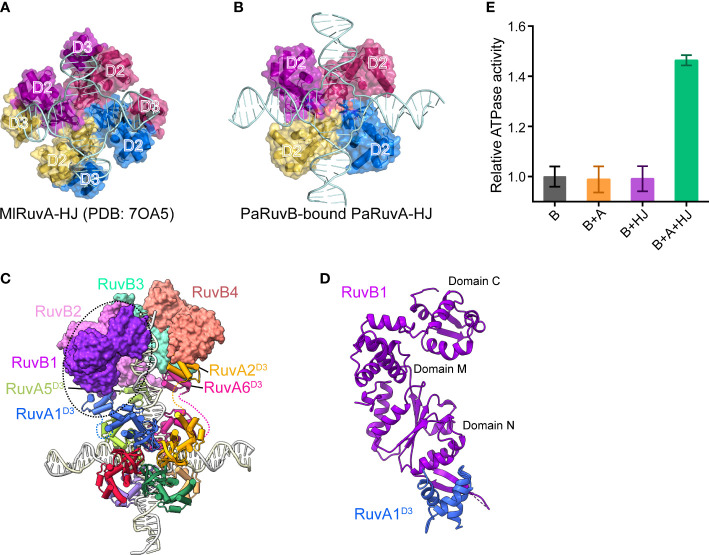
Recruitment and assembly of the RuvB motor. **(A, B)** Domain arrangement of RuvA in the crystal structure of MlRuvA-HJ complex (PDB: 7OA5) **(A)** and in the cryo-EM structure of RuvAB-HJ complex **(B)**. RuvA subunits are shown in surface with various colors. The RuvA^D2^ and RuvA^D3^ domains are labeled. Ml, *Mycobacterium leprae;* Pa, *Pseudomonas aeruginosa*. **(C)** Model of RuvAB-HJ complex. RuvB subunits are shown in surface and in different colors. RuvA subunits are shown in cartoon. HJ DNA is in ladder representation. **(D)** The interaction between PaRuvB (violet) and PaRuvA^D3^ (blue). **(E)** Measurements of RuvB ATPase activities in the presence of RuvA, HJ or both (mean ± s.d., n = 3 independent experiments).

Structural comparison between the current cryo-EM structure of PaRuvAB-HJ complex and the previous MlRuvA-HJ complex (PDB ID: 7OA5)(Roe et al., 1998) revealed a prominent rearrangement of RuvA^D3^ (**Figures 4A, B**). In the absence of RuvB, MlRuvA^D3^ packs against MlRuvA^D2^ of the neighboring subunit, resulting in severe steric clashes with HJ duplex arms (**Figure 4A**). In the current structure, however, all the eight PaRuvA^D3^ spring out from the junction center and each individually captures a PaRuvB subunit (**Figures 4B–D**). As a result, the HJ DNA molecule can be perfectly docked onto RuvA without steric hindrance (**Figure 4B**). Consistent with these observations, while RuvA and HJ each alone had no effects on the ATP hydrolysis by RuvB, a combination of the two significantly stimulated its activity (**Figure 4E**), suggesting that RuvB alone can not for a functional closed hexameric ring onto HJ.

**Figure 5:**
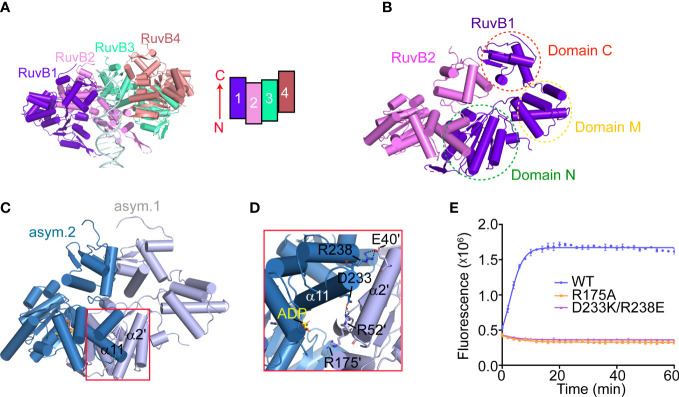
The packing pattern adopted by PaRuvB subunits in the cryo-EM structure. **(A)** Cartoon representation of the RuvB-dsDNA complex. α-helices are shown in cylinder. DNA duplex is shown in ladder representation. The relative orientation and packing pattern of each RuvB subunit are illustrated on the top-left. **(B)** The dimerization between RuvB1 and RuvB2 as shown in **(A)** The three domains (N, M and C) of RuvB are indicated. **(C)** The dimerization interface of RuvB subunits revealed by crystal packing. **(D)** Close-up view of the RuvB intersubunit binding region defined in **(C)**. Key interacting residues are shown in sticks and labeled. Dashed lines indicate electrostatic interactions. **(E)** Effects of indicated mutations on the HJ branch migration activities of RuvAB by FRET-based assay. Values are means ± s.d., n = 3 independent experiments.

To reconstruct the 3D model of PaRuvB-dsDNA complex, we determined a 2.1-Å crystal structure of PaRuvB in complex with ADP. The crystal structure of PaRuvB-ADP complex is essentially identical to the *Thermotoga Maritima* (*T. Maritima*, Tm) RuvB (PDB ID: 1IN4), which consists of three consecutive domains (N, M and C) and contains canonical AAA+ ATPase fold at domain N (**Supplementary Figure S5**). In the cryo-EM structure of PaRuvB-dsDNA complex, the four PaRuvB subunits pack against with each other using a binding interface similar to that used for crystal packing (**Figures 5A–C**). The intersubunit interaction involves E40 and R52 on α2 of one subunit and D233 and R238 on α11 of the other subunit (**Figure 5D**). In particular, the arginine finger R175 (equivalent to R174 of EcRuvB) is located on this interface and approaches to the β-phosphate of ADP in the neighboring subunit (**Figure 5D**). Charge-reversal mutations of D233/R238 or alanine substitution of R175 eliminated the branch migration activity (**Figure 5E**). These results thereby demonstrate that the intersubunit interface of RuvB observed here is functionally related.

### 2.5 Structure of the PaRuvB-dsDNA complex

**Figure 6:**
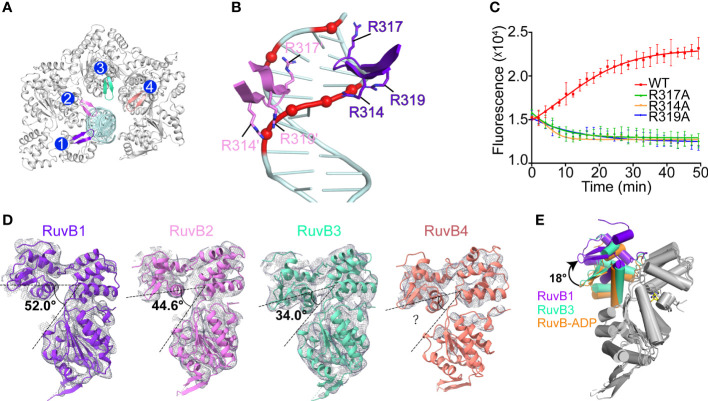
The interactions between PaRuvB and DNA. **(A)** Top-view of the structure of the RuvB-DNA complex. The DNA interacting elements of RuvB are highlighted in different colors. **(B)** Zoomed in view of the interactions between RuvB and DNA duplex. Key RuvB residues and DNA backbone phosphates are shown in sticks and spheres, respectively. **(C)** Effects of RuvB mutations on the branch migration activity of RuvAB complex (mean ± s.d., n = 3 independent experiments). **(D)** Conformational variations of RuvB subunits in the cryo-EM structure. RuvB subunits are aligned on their N-terminal domains and overlaid with 2mFo-DFc map contoured at 0.28 σ. The angle between domains N and C is defined by the Cα atoms of G165-V186-T314. **(E)** Comparison of RuvB subdomain orientations between the cryo-EM structure and the ADP-bound crystal structure (orange). RuvBs are aligned on their N-terminal domains. ADP is shown in yellow stick.

The four PaRuvB subunits form an asymmetric spiral and encircle 22 bp of HJ arm, and two of them intimately contact the DNA minor groove with their β-hairpins in domain C, involving resides R314, R317 and R319 (**Figures 5A**, **6A, B**). Alanine substitution of R314, R317 or R319 on the β-hairpin abolished the branch migration activity of PaRuvAB complex (**Figure 6C**), suggesting that they are involved in the interactions with DNA. Furthermore, because the β-hairpin is in close proximity to both DNA strands, we propose that R317 may bind a backbone phosphate from the first strand, while R314 and R319 may interact with two consecutive phosphates from the second strand (**Figure 6B**). This suggests that each PaRuvB translocates 2 bp of DNA per catalytic step, and for a full cycle within the intact RuvAB-HJ complex that containing 6 RuvB, 12 bp of DNA is translocated through the hexameric ring.

Structural analysis revealed distinct subdomain orientations of the four PaRuvB subunits. The angle between domains N and C defined by the Cα atoms of G165-V186-T314 increased from 34° for RuvB3 to 52° for RuvB1 (**Figure 6D**). The conformation for RuvB4 could not be unambiguously defined due to the poor electron density on the N-terminal part. Such gradual conformational changes have been correlated to different nucleotide states in other ring helicases (Enemark and Joshua-Tor, 2006; Thomsen and Berger, 2009; Jean et al., 2020). Due to the limited resolution of current cryo-EM structure, the nucleotide state for each PaRuvB subunit could not be immediately assigned. Despite that, structural comparisons showed that the overall conformations of RuvB3 and RuvB4 are close to the crystal structure of ADP-bound RuvB (**Figure 6E**), implying that these two DNA-free subunits are probably in ADP-bound state. Furthermore, the subunits involved in nucleic acid substrate binding are generally bound by ATP in the reported structures of hexameric helicases (Enemark and Joshua-Tor, 2006; Thomsen and Berger, 2009; Jean et al., 2020). Thus, the two subunits RuvB1 and RuvB2 are likely in ATP-bound state. We propose that DNA binding would trigger ATP hydrolysis and subdomain motion in RuvB, which in turn drives DNA translocation from domain N to domain C.

## 3 Discussion

In this study, we have reconstituted the PaRuvAB-HJ tripartite complex *in vitro* and determined its structure by cryo-EM. The structure reveals an unusual assembly pattern of RuvB subunits. The eight RuvB subunits constitute two open-rings encircling two opposite HJ arms, and each RuvB subunit binds to a RuvA^D3^, suggesting an intermediate state of the RuvAB-HJ complex.

**Figure 7:**
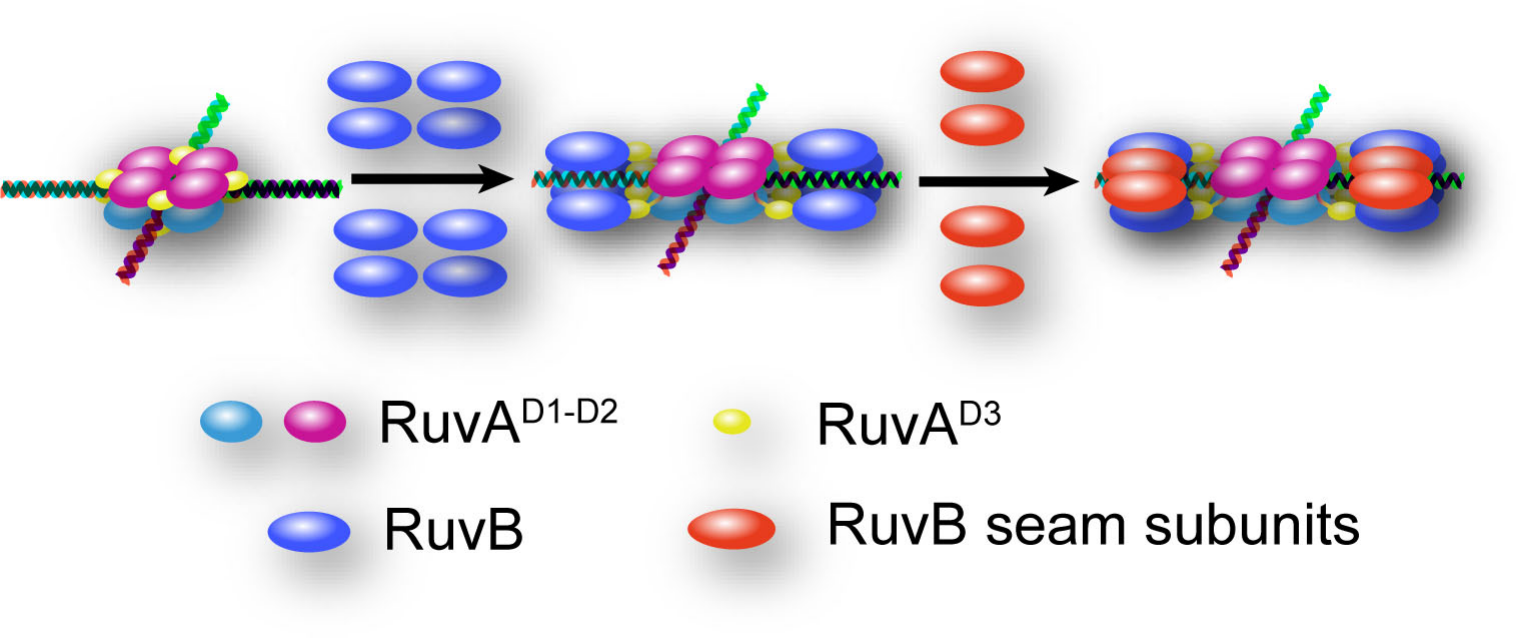
Hypothetical model for the mechanism of RuvB motor assembly onto HJ DNA. HJ DNA is depicted as four-way double stranded helix. RuvA^D1-D2^ of the top and bottom tetramers are colored in magenta and cyan, respectively. All RuvA^D3^ are colored in yellow. The eight RuvB subunits initially loaded as open rings are colored in blue, and the four RuvB seam subunits are shown in red.

While we were preparing this manuscript, Wald et al. reported the cryo-EM structures of hetero-RuvAB complex bound to HJ, providing unprecedented details of how ATP hydrolysis and nucleotide exchange drive HJ branch migration (Wald et al., 2022). In the structure, the RuvB hexamer is assembled in a closed ring shape. Only one RuvA tetramer binds to the RuvB hexameric motors with two RuvA^D3^ on each side. Based on these structures and our intermediate structure of RuvAB-HJ complex, we propose that RuvB motor may initially be loaded as four-subunit open ring, with each subunit attaching to a single RuvA^D3^ from the RuvA octamer core, then the gap between the two ends of the open ring could be filled by another two RuvB subunits (seam subunits) (**Figure 7**). Similar mechanisms have been reported for the assembly of archaeal MCM and eukaryotic CMG helicases, where five subunits within MCM and three or four subunits within CMG form spirals, and the remaining seam subunits bridge the ends of each spiral (Georgescu et al., 2017; Goswami et al., 2018; Eickhoff et al., 2019; Meagher et al., 2019). These structures together with the biochemical analyses implicate a potential mechanism of RuvB motor assembly onto HJ DNA, which is important for the ultimate understanding of how RuvAB complex catalyzes homologous recombination.

## 4 Materials and methods

### 4.1 Protein expression and purification

The cDNA encoding PaRuvA (GenBank ID: 879609) and RuvB (GenBank ID: 882028) proteins were synthesized at GenScript (Nanjing, China) and subcloned into pGEX-6P-1 bacterial expression vector containing an N-terminal glutathione S-transferase (GST) tag. The recombinant vectors containing RuvA and RuvB genes were transformed into *E. coli* strain Rosetta (DE3) and BL21 (DE3) competent cells, respectively. Cells were grown in the LB media at 37°C until the OD_600_ reached around 0.8. Protein expressions were induced with 0.5 mM isopropyl-β-D-1-thiogalactopyranoside (IPTG) at 17°C overnight. The purification of RuvA and RuvB proteins was performed as previously described(Lin et al., 2019). Briefly, cells were resuspended with lysis buffer (50 mM HEPES pH 7.4, 200 mM NaCl, 5% (v/v) glycerol and 1 mM dithiothreitol (DTT)) and disrupted by high pressure homogenizer (Union Biotech, China). The GST-tagged proteins in the supernatant of cell lysates were pooled through a GST Sepharose column (Senhui Microsphere Technology, China). The GST tags were subsequently removed by home-made PreScission proteases and the untagged proteins were further purified using Source 15Q and Superdex 200 10/300 GL columns (GE Healthcare Life Sciences) in running buffer containing 20 mM HEPES pH 7.4, 200 mM NaCl, 15 mM reduced glutathione and 1 mM DTT. The purified proteins were concentrated, aliquoted and stored at -80°C. The mutant variants of RuvA and RuvB were expressed and purified similarly.

### 4.2 HJ DNA substrates preparation

All of the oligonucleotides used in this study (**Supplementary Table S1**) were synthesized and HPLC-purified by SunYa Biotechnology (Fuzhou, China), and dissolved in annealing buffer (100 mM HEPES pH 7.4, 50 mM NaCl). For HJ DNA annealing, the four HJ strands were divided into two groups, A/B and C/D. Each group of DNA strands were subjected to heating at 95°C for 5 min, followed by gradual cooling to room temperature. Thereafter, the annealed products of both groups were mixed and incubated at 37°C for 30 min, and then cooled down to room temperature. For DNA imaging, one strand of HJ was labeled with 6-carboxyfluorescein (FAM). For FRET-based measurement, two complementary strands of HJ were labeled with Cyanine 3 (Cy3) and Black Hole Quencher^®^ 2 (BHQ2), respectively.

### 4.3 Luciferase-based ATPase assay

The ATPase activity of RuvB was determined by a firefly luminescence assay as described previously (He et al., 2022). Briefly, in 100 μl reaction, various concentrations of RuvB proteins were mixed with reaction buffer containing 50 mM Tris-HCl pH 7.4, 100 mM NaCl, 10 mM MgCl_2_, 1 mM DTT, 0.1% Tween20. Reactions were initiated by the addition of 10 μM ATP, and further incubated at 37°C for 1h. Subsequently, the reactions were stopped by addition of Kinase-Glo reagent (Promega), and the residual amounts of ATP were measured by luminescence on the SpectraMax-L microplate reader (Molecular device).

### 4.4 HJ branch migration assay

The branch migration assay was performed as previously described (Hishida et al., 1999; Dawid et al., 2004). Briefly, 20-μl of reactions containing 250 nM FAM-labeled HJ DNA substrates (HJ1, **Supplementary Table S1**) and various concentrations of RuvA and RuvB proteins in branch migration buffer (50 mM Tris pH 8.0, 10 mM MgCl_2_, 20 mM NaCl, 1 mM DTT, 1mM ATP, 0.1 mg/ml BSA) were incubated at 37°C for 60 min. The reactions were terminated by adding 2 μl of proteinase K (2 mg/ml) and 5 μl of 5× stop buffer (100 mM Tris pH 8.0, 250 mM EDTA, 10% (w/v) SDS, 5% (v/v) glycerol and 0.1‰ (w/v) bromophenol blue), followed by incubation at 37°C for 15 min. The products were analyzed by 10% TBE-native PAGE (110 V, 40 min) and visualized with the ChemDoc™ touch imaging system (Bio-Rad, USA). For FRET-based measurement of branch migration activities, in 50-μl aliquots, 250 nM Cy3/BHQ2-labeled HJ DNA substrates were mixed with 800 nM RuvA, 1200 nM RuvB, or indicated protein concentrations, in branch migration buffer. Reactions were initiated with 1 mM ATP and incubated at 37°C. The fluorescence was recorded every 2 min using a SpectraMax i3x plate reader (Molecular Devices, USA) with λ_ex_/λ_em_ = 542 nm/567 nm.

### 4.5 Crystallization, data collection and structure determination

For crystallization of RuvB-ADP complex, RuvB (10 mg/ml) was pre-incubated with 10 mM ADP in 20 mM HEPES pH 7.4, 200 mM NaCl, 10 mM MgCl_2_, and 1 mM DTT. The protein solution was mixed with an equal volume of precipitant solution containing 0.2 M L-Proline, 0.1 M HEPES pH 7.5, 10% (w/v) polyethylene glycol 3350 (PEG3350) and 10 mM Trimethylamine hydrochloride. Crystals were grown under 18°C using the sitting drop vapor diffusion method. Diffraction-quality crystals were fast-frozen in liquid nitrogen with reservoir solution supplemented with 15-25% (v/v) glycerol. X-ray diffraction data were collected at beamline BL19U1 at Shanghai Synchrotron Radiation Facility (SSRF) with a wavelength of 0.978 Å, and processed with HKL3000(Minor et al., 2006). The crystal structure of RuvB-ADP complex was solved by molecular replacement with the program Phaser-MR in the PHENIX package (Liebschner et al., 2019), using the structure of *T. Maritima* RuvB (PDB ID: 1IN4) as a searching model. Iterative manual model building and structural refinements were conducted using the programs of Phenix (Liebschner et al., 2019) and WinCoot (Emsley et al., 2010). The final model was validated by MolProbity (Chen et al., 2010). Data collection and structure refinement statistics were summarized in **Supplementary Table S2**.

### 4.6 Cryo-EM sample preparation and image acquisition

To reconstitute RuvAB-HJ complex, HJ DNA substrates (HJ2, **Supplementary Table S1**) in 50 mM NaCl, 20 mM HEPES pH 7.4, 1 mM ATPγS, 10 mM MgCl_2_ and 1 mM DTT were pre-incubated with RuvA at 4°C for 15 min, and then further incubated with RuvB for another 15-min, with a final molar ratio of RuvA:RuvB:HJ = 8:16:1.2. The RuvAB-HJ complex was further purified through a Superose 6 10/300 GL column (GE Healthcare Life Sciences) equilibrated in buffer containing 20 mM HEPES pH 7.6, 100 mM NaCl, 10 mM MgCl_2_ and 1 mM DTT. The corresponding fractions were pooled and concentrated to about 4 mg/ml. Samples were subjected to negative staining for homogeneity analysis prior to cryo-EM experiments. The cryo-EM grids were prepared by applying 2 μl aliquot of the sample to glow-discharged Quantifoil™ R1.2/1.3 300 mesh gold grids. The grids were blotted for 3 s at force-3 before being plunged into liquid ethane using a Vitrobot Mark IV (Thermo Fisher Scientific) operated at 4°C and 100% humidity. Grids were transferred to an FEI Titan Krios (Thermo Fisher Scientific) operated at 300 kV high tension and images were collected semi-automatically with SerialEM 4.0 software under low-dose mode at a nominal magnification of 130,000×, with a resulting pixel size of 0.83 Å. A Gatan K3 Bioquantum summit direct electron detector was used under super-resolution mode for image recording with defocus values ranged between -3.0 and -1.0 µm, and a total accumulated dose of 60 e^-^/Å^2^ on the specimen. Detailed information was summarized in **Supplementary Table S3**.

### 4.7 Cryo-EM data processing

Image processing was performed using the software packages RELION 3.1 (Zivanov et al., 2020) and cryoSPARC 3.1 (Punjani et al., 2017), and summarized in a flowchart in **Supplementary Figure S2**. A total of 5,535 movie stacks were subjected to beam-induced motion correction using MotionCor2 in Relion 3.1 (Zheng et al., 2017), and their contrast transfer function (CTF) parameters were estimated with CTFFIND4 in cryoSPARC (Rohou and Grigorieff, 2015). About 2,000 particles from different views were manually picked and 2D classified. Using these 2D classes as references, a total number of 584,587 particles were automatically picked and extracted (box size 480 pixels) from denoised images with Topaz in cryoSPARC (Bepler et al., 2019; Bepler et al., 2020). All the particles were then 2D classified and the unclassified were removed. After 2D classification, 551,160 particles appeared as dumbbell shape were selected and sorted into four classes through Ab-Initio reconstruction and heterogeneous refinement. Subsequently, 143,397 particles with the coordinate information were selected and re-extracted by Relion 3.1 and further improved using 3D refinement, post-processing, Bayesian polishing, and homogeneous refinement, resulting in an overall density map with a resolution of 3.21 Å. The refined particles and a soft mask focused on RuvA region were used to build the final density map of RuvA region with a resolution of 3.01 Å, as determined by Fourier shell correlation (FSC) using the correlation cutoff value of 0.143 (Chen et al., 2013). To probe the conformational dynamics and improve the resolution of RuvB region, 101,792 Bayesian polished particles corresponding to RuvAB-HJ complexes were subjected to another round of 3D focused classification without alignment in Relion 3.1 and 3D variability analysis in cryoSPARC (Punjani and Fleet, 2021), respectively. Particle subsets with improved density in RuvB region were selected after 3D skip alignment classification (25,482 particles) and 3D variability analysis (29,607 particles), and the duplicate particles (16,258 particles) were removed by particle sets tool in cryoSPARC. After another round of heterogeneous refinement, 20,536 particles were selected for final homogeneous refinement with the application of a soft mask, yielding an overall density map for RuvAB-HJ complex with a resolution of 6.18 Å. After local refinement with a mask of RuvB region and map sharpening with a negative B factor of -300 Å^2^, the resolution of density map within the RuvB region defined by the soft mask is estimated to be 7.02 Å.

### 4.8 Structural modeling, refinement, and validation

The RuvA-HJ model was built with a 3.01-Å-resolution map using the crystal structure of *E. coli* RuvA-HJ (PDB ID 1C7Y) (Ariyoshi et al., 2000) as template. The model of RuvB-dsDNA complex was built with a cryoEM map of 7.02 Å, using the crystal structure of RuvB-ADP complex determined in this study. Each subunit was manually fitted into the density map in UCSF Chimera 1.15 (Pettersen et al., 2004), and then refined using Phenix(Liebschner et al., 2019) and WinCoot (Emsley et al., 2010) and the ISOLDE flexible atomic model refinement package in UCSF ChimeraX 1.2.5 (Croll, 2018). The resulting models of RuvA-HJ and RuvB-dsDNA complexes were used to build a composite model of RuvAB-HJ complex using UCSF Chimera 1.15, which was refined with a similar procedure mentioned above. The final models were finely adjusted for geometry violations guided by electron density. Cryo-EM data collection, reconstruction and refinement statistics and the final refinement statistics were summarized in **Supplementary Table S3**. All structural figures in this paper were generated with UCSF ChimeraX 1.2.5 (Goddard et al., 2018) and PyMOL (http://www.pymol.org/).

## Data availability statement

The datasets presented in this study can be found in online repositories. The names of the repository/repositories and accession number(s) can be found below: https://www.ebi.ac.uk/pdbe/emdb/, EMD-33044, EMD-33043, EMD-33013; http://www.wwpdb.org/, 7X7Q, 7X7P, 7X5A, 7X5B.

## Author contributions

XZ conducted all the biochemical experiments, processed the cryo-EM data and built the initial structural model. ZZ built and refined the cryo-EM structures. YC assisted with cryo-EM grid screening. LD crystallized the RuvB-ADP complex. ZLin, QQ, and MH supervised the project. XZ and ZLin wrote the paper. All authors contributed to the article and approved the submitted version.
