# Supplementary material for: Cryo-EM structure of the RuvAB-Holliday junction intermediate complex from Pseudomonas aeruginosa
Source: Front Plant Sci. 2023 Mar 21;14:1139106. doi: 10.3389/fpls.2023.1139106 (PMC10071043; doi:10.3389/fpls.2023.1139106)
Supplement: Supplementary file 1 [file DataSheet_1.pdf]

Supplementary Material

Cryo-EM structure of the RuvAB-Holliday junction in-termediate complex from *Pseudomonas aeruginosa*

Xu Zhang*, Zixuan Zhou*, Qianhui Qu, Zhonghui Lin

*** Correspondence:** Qianhui Qu: qqh@fudan.edu.cn; Zhonghui Lin: zhonghui.lin@fzu.edu.cn

1 Supplementary Figures and Tables

1.1 Supplementary Figures

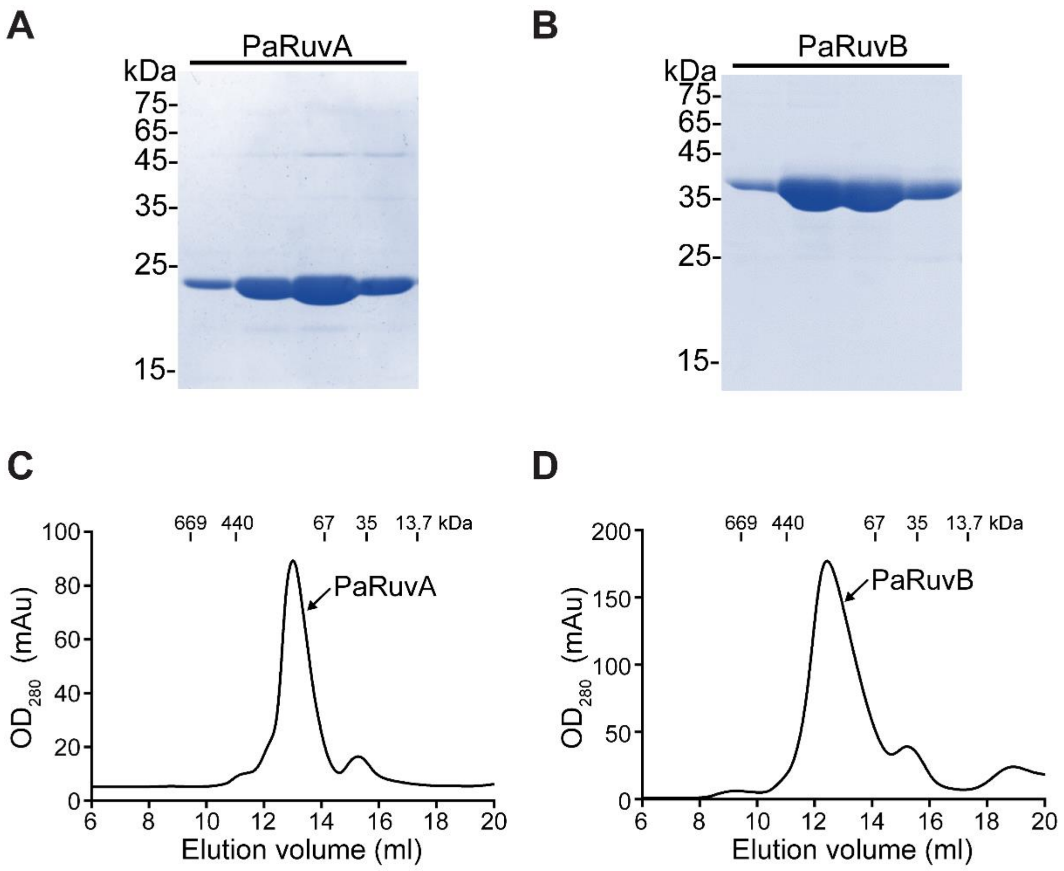

Figure S1. Purification of PaRuvA and PaRuvB recombinant proteins. **(A, B)** Coomassie-stained gels of the purified PaRuvA and PaRuvB proteins. **(C, D)** Size exclusion chromatography spectra of the PaRuvA and PaRuvB proteins.

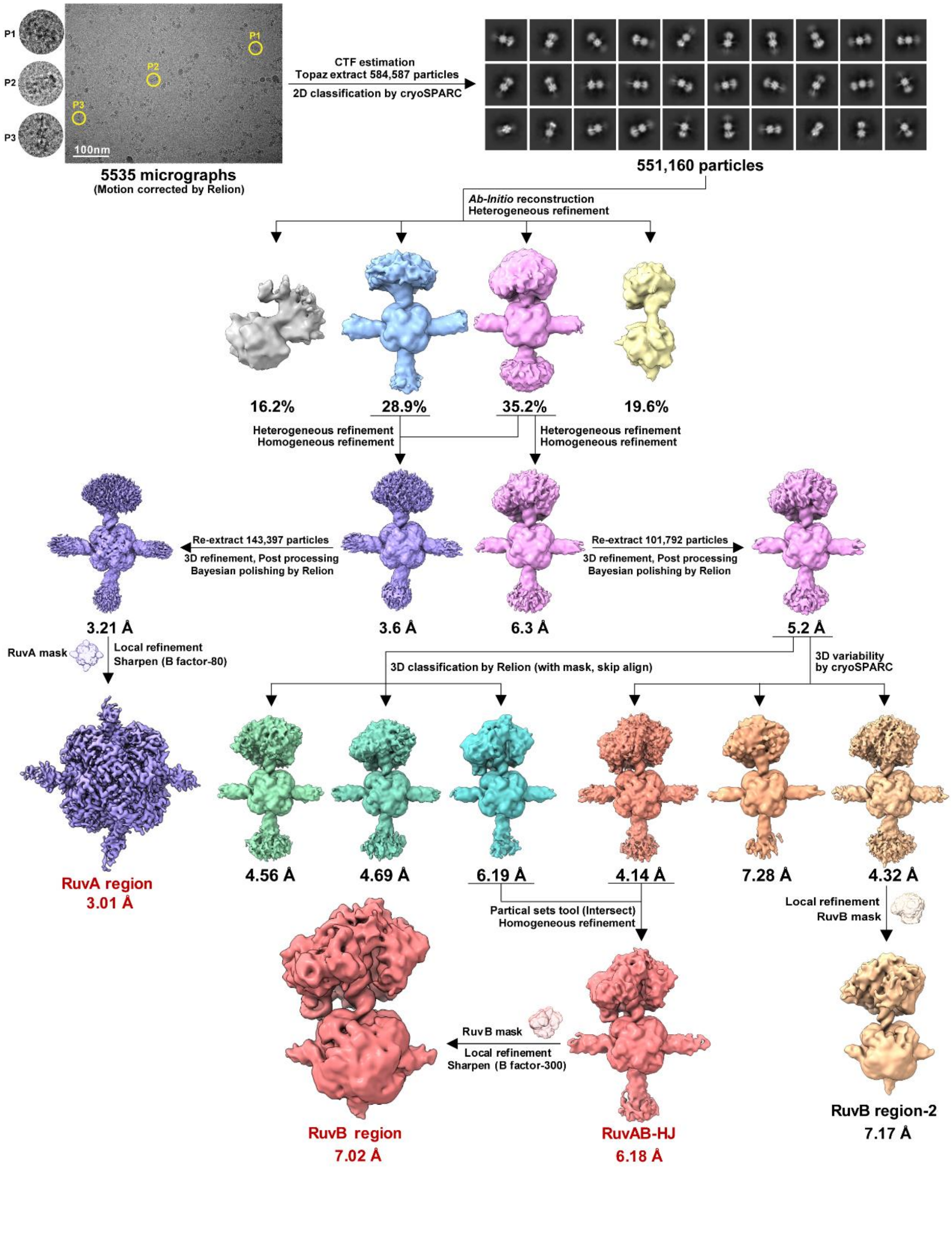

Figure S2. Flowchart of cryo-EM data processing. See also the Materials and Methods section for a detailed description. The resolutions were estimated on the basis of the Fourier shell correlation (FSC) value of 0.143. The mask used for sharpening was also used for global resolution determination.

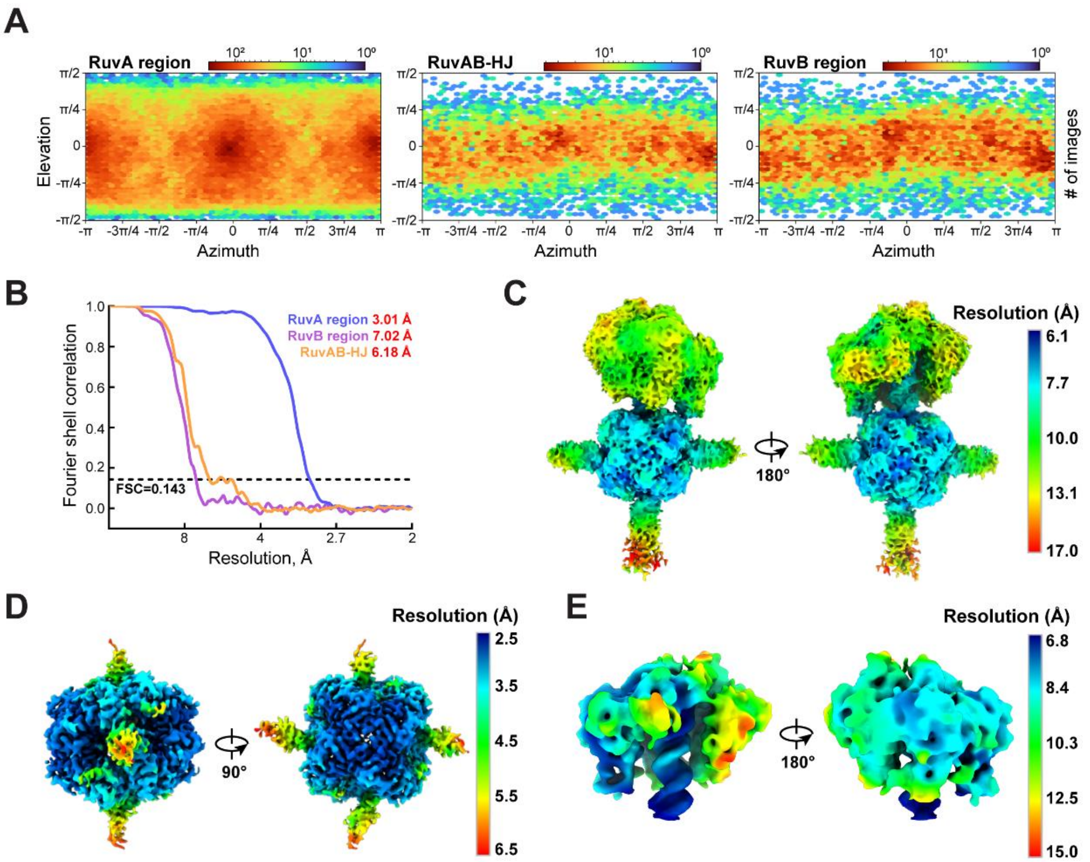

Figure S3. Cryo-EM map resolution and validation. **(A)** Euler angle distributions of the particle sets used in the final maps. **(B)** Fourier shell correlation (FSC) curves of the final density maps. **(C-E)** Local resolution estimations of the density maps of RuvAB-HJ, RuvA-HJ and RuvA domain III-RuvB-dsDNA complex as calculated by cryoSPARC 3.1.

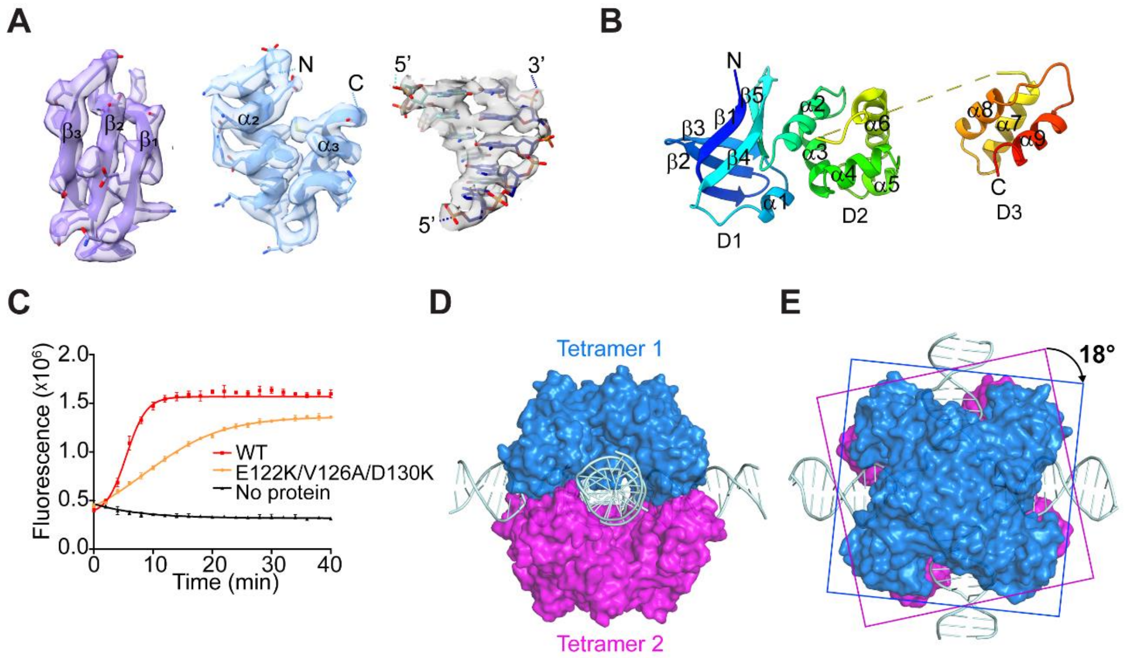

Figure S4. Cryo-EM structure of RuvA-HJ complex. **(A)** Local densities of representative regions for RuvA and HJ DNA. **(B)** Cartoon representation of PaRuvA monomer. Secondary structure elements are labeled. The N and C termini are indicated. Segment of the peptide with no visible electron densities is indicated as broken line. **(C)** Effects of indicated RuvA mutations on the branch migration activity of RuvAB complex by FRET-based measurement. **(D)** Side-view of RuvA-HJ complex, with two RuvA tetramers shown as blue and magenta surface, and DNA shown as ladder. (e) Top-view of RuvA-HJ complex. The two tetramers do not form a perfect two-fold symmetry but are misaligned by ~18°.

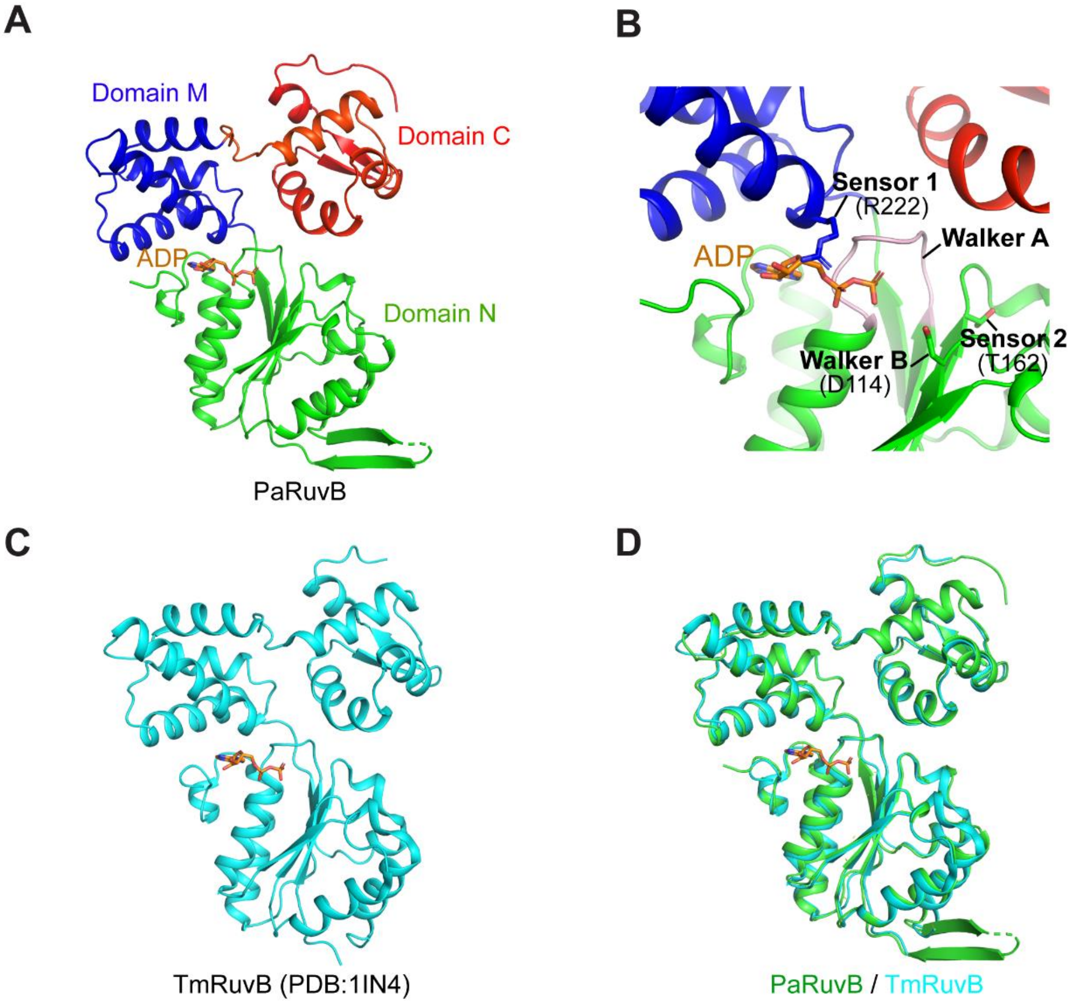

Figure S5. Crystal structure of PaRuvB. **(A)** Cartoon representation of the crystal structure of PaRuvB, with each domains colored and labeled. **(B)** Key sensors and motifs within the catalytic site of PaRuvB. **(C)** Cartoon representation of the crystal structure of TmRuvB. **(D)** Structural alignment between PaRuvB and TmRuvB. Pa, *Pseudomonas aeruginosa*; Tm, *Thermotoga Maritima*.

1.2 Supplementary Tables

Table S1. Oligos used in this study

Name	Sequence 5'→3'	Labels
Oligos used for gel-based HJ migration assay		
HJ1-A	CAC TGT GAT GCA CGA TGA TTG ACG ACA GTA GTC AGT GCT GCA GTG GTC AGG TGT C	5'-FAM
HJ1-B	ATA CAG ATG TTG ACC CAG CAC TGA CTA CTG TCG TCA ATC ATC GTG CAT CAC AGT G	
Oligos used for FRET-based HJ migration assay		
HJ1-A	CAC TGT GAT GCA CGA TGA TTG ACG ACA GTA GTC AGT GCT GCA GTG GTC AGG TGT C	5'-Cy3
HJ1-B	ATA CAG ATG TTG ACC CAG CAC TGA CTA CTG TCG TCA ATC ATC GTG CAT CAC AGT G	3'-BHQ2
Oligos used for both gel- and FRET-based HJ migration assays		
HJ1-C	CAC TGT GAT GGC ATG AAT CCG ACG ACA GTA GTC AGT GCT GGG TCA ACA TCT GTA T	
HJ1-D	GAC ACC TGA CCA CTG CAG CAC TGA CTA CTG TCG TCG GAT TCA TGC CAT CAC AGT G	
Oligos used for the reconstitution of RuvAB-HJ complex for cryo-EM analysis		
HJ2-A	CAC TGT GAT GCA CGA TGA TAG AAC ACA GTA CTC AGT GCT GCA GTG CTC AGG TGT G	
HJ2-B	ATA CAG ATG TAG AGC CAG CAC TGA GTA CTG TGT TCT ATC ATC GTG CAT CAC AGT G	
HJ2-C	CAC TGT GAT GGC ATG TAT CCG AAC ACA GTA CTC AGT GCT GGC TCT ACA TCT GTA T	
HJ2-D	CAC ACC TGA GCA CTG CAG CAC TGA GTA CTG TGT TCG GAT ACA TGC CAT CAC AGT G	

Table S2. X-ray diffraction data collection and refinement statistics

**Data collection**	
Space group	P65
Wavelength	0.978
Cell dimensions	
a, b, c (Å)	85.677, 85.677, 76.973
α, β, γ (°)	90, 90, 120
Resolution range (Å)	50-2.1 (2.18-2.10)
Redundancy	20 (18)
Data completeness (%)	99.6 (97.1)
R_{merge}	0.14 (1.48)
I/σ (I)	23.9 (2.6)
**Refinement**	
Resolution (Å)	24.7- 2.2 (2.24- 2.16)
No. Reflections	17197 (1667)
R_{factor} / R_{free}	0.173 / 0.196
No. atoms	2663
Macromolecules	2440
Ligand	27
Solvent	196
B-factor	27.14
Macromolecules	26.70
Ligand	26.45
Solvent	32.68
RMSD bond length (Å)	0.008
RMSD bond angles (°)	1.21
Ramachandran Favoured (%)	
Favoured/allowed/disallowed	98.7 / 1.3 / 0

Table S3. Cryo-EM data collection, reconstruction and refinement statistics

**Data collection and processing**			
EM equipment		FEI Titan Krios	
Voltage (kV)		300	
Magnification		130,000	
Detector		Gatan K3 Bioquantum	
Spherical aberration (mm)		2.7	
Electron exposure (e^-/Å^2)		60	
Defocus range (µm)		-1.0 ~ -3.0	
Pixel size (Å)		0.83	
Total number of images		5535	
Software		SerialEM 4.0/RELION 3.1/cryoSPARC 3.1	
Data set	RuvA-HJ	RuvAB-HJ complex	RuvB-dsDNA
Number of used particles	143,397	20,536	20,536
Final resolution (Å)	3.01	6.18	7.02
FSC threshold	0.143	0.143	0.143
Map sharpening B factor (Å^2)	-80	-300	-300
**Refinement**			
Protein residues	1083	2511	1428
Nucleotides	104	132	46
Nonhydrogen atoms	10686	22135	11776
RMSD Bond lengths (Å)	0.003 (0)	0.004 (1)	0.005 (0)
RMSD Bond angles (°)	0.545 (0)	0.851 (4)	0.976 (10)
MolProbity score	1.69	1.82	1.87
Clash score	3.69	11.68	11.59
Ramachandran plot (%)			
Favoured/Allowed/Outliers	98.31/1.69/0.00	96.44/3.56/0.00	95.80/4.20/0.00
Rotamer outliers (%)	0.00	0.10	0.18
Cβ outliers (%)	0.00	0.00	0.00
**PDB ID**	7X5A	7X7Q	7X7P

1.3 Supplementary Movie

Movie S1. The 3D variability analysis of the cryo-EM map of RuvAB-HJ complex.
